# Mitigating Health Risks After Floods and Landslides in Nepal: A Public Health Perspective

**DOI:** 10.3126/nje.v14i4.83897

**Published:** 2025-09-01

**Authors:** Padam Simkhada, Brijesh Sathian, Bibha Simkhada, Edwin van Teijlingen, Pragya Koirala

**Affiliations:** 1 School of Human and Health Sciences, University of Huddersfield, Huddersfield, United Kingdom; 2 Geriatrics and long-term care Department, Rumailah Hospital, Hamad Medical Corporation, Doha, Qatar; 3 Centre for Midwifery & Women’s Health, Bournemouth University, Bournemouth, United Kingdom; 4 Senvers, Queensland, Australia

## Background

September 2024 Nepal experienced a high intensity natural calamity due to excessive rainfall and flooding with Landslides. At least 230 people, including 55 children, are believed to have been killed, and many more have been injured or remain missing. More than 10,000 homes lost or damaged and these fires displaced around 50,000 people. Critical infrastructure, including 62 health facilities, 550 water supply systems and 9,000 sanitation facilities, has been knocked down. More than 190 schools have been vandalized, impacting over 23,000 school children [1]. Landslides and debris obstructed major roads that snake into the Katmandu Valley, and put a halt to flights in and out of Tribhuvan International Airport in Kathmandu, resulting in severely restricted access to important commodities and vital services.

The disruption of roads, drinking water supply, sewer systems, and population displacement has worsened the public health situation with the health and wellbeing of the affected communities severely at risk. Disasters, such as this one, result in short and long-term public health crises including disease outbreak, mental health effects, and socio-economic effects.

In the immediate aftermath, the catastrophe raises the risk of epidemics of waterborne diseases such as acute diarrhoeal diseases (including cholera), vector- borne diseases, skin infections and respiratory ailments. There were flooded houses where sanitation infrastructure was damaged resulting in surface water pollution creating a conducive environment for pathogens to spread. Monstrous disasters of this nature in South Asia have caused widespread outbreaks of cholera and diarrhoeal diseases in the past, owing to the destruction of clean water sources [2]. Additionally, pooled water, combined with hot post-disaster temperatures, can also result in new outbreaks of vector-borne diseases like malaria and dengue fever.

The medium- and long-term consequences for health are also alarming, especially for the mental health. Some survivors will suffer from different mental health problems, such as post-traumatic stress disorder (PTSD), depression and anxiety, just as communities will grapple with the trauma of the aftermath and fear of the unknown (loss, dislocation/ homelessness) [3]. Five years ago, we reported on the rise of post-traumatic stress disorders [4]. These mental health ailments can be exacerbated by the slow economic and social recovery.

**Strengthening Disease Surveillance Systems:** The government, together with public health organisations, needs to improve surveillance of diseases to more quickly identify and address outbreaks. An early recognition of cholera, diarrhoeal diseases and vector-borne disease can prevent widespread epidemics. Mobile health teams and quick response teams could also be stationed at the affected locations, and the health needs assessed better and faster, and treatment provided on the spot.

**Restoring Water, Sanitation, and Hygiene (WASH) services:** Available now, but not yet happening at scale; the most urgent action includes prioritizing the restoration of access to safe water and operational sewage systems to avoid further exacerbating the spread of waterborne diseases. Normal treatment of water (chlorine tablets) and clear drinking water plus hygiene kits (as preventive measures against contamination) are important factors in reducing the cases of diarrhoea and other infectious diseases.

**Health Promotion and Community Mobilization:** Public health interventions that promote the practice of good hygiene such as handwashing, and the proper disposal of waste are of utmost importance. Community education on strategies for reducing vector exposure and preventing contamination of water sources enables the community to take measures to prevent the disease.

**Mental Health and Psychosocial Support (MHPSS):** Attention to mental health is important during recovery. Building the mental health system and training responders in psychological/mental health first aid will in turn support survivors when they are processing the trauma and beginning to recover. Studies show the efficacy of community-based interventions in promoting mental health in the aftermath of disasters.

**Rebuilding Broken Health Systems:** The health facilities in disasters-affected regions should be constructed to withstand the flooding in the first place; also be equipped to manage the increased number of patients. Temporary health posts can be reinstated to treat immediate injuries and prevent the spread of disease, as well as the long-term provision of mental health care.

**Disaster Risk Reduction (DRR) Strategy:** Nepal needs to further invest in disaster preparedness and build resilience, including updating early warning systems, constructing in-flow defences, planting trees that can store water in the soil, and preparing health systems for any potential health emergencies created by natural disasters [6]. Inclusion of public health in DRR will enhance the country’s resilience to future events.

Resilient waste collection and disposal - During periods of flooding, waste that is abandoned or left exposed can result in groundwater pollution, contamination and public health threats, and it is necessary to allocate landfills with an elevation with composite lining and liquid-waste recovery to prevent groundwater from being contaminated. Additionally, implementation of sturdy fencing and containment facilities is needed in order to avoid waste spreading into surface waters during flood events. Moreover, the use of regular maintenance practices and the enhancement of waste separation or segregation in drainage network via community-based waste management programs are highly recommended, in that these measures can significantly reduce blockages and enhance flood resistance. Local authorities should also make sure they also embrace sustainable urban drainage options such as swales, retention basins, and created wetlands that could similarly minimize the flood risk and deliver ecological benefits.

**Reinforce water pollution prevention and sanitary measures:** The frequency of floods is going up, which raises the risk of waterborne diseases from contaminated water sources. Mitigation measures should comprise the construction of protective infrastructure around water bodies, together with the assistance of hazardous waste containment to avoid the entry of pollutants to the aquatic ecosystem. Promoting waste recycling and segregation through public education and providing accessible recycling centres is necessary to reduce waste accumulation. In this manner the likelihood of blockages of flow passages is reduced and contamination is minimized. Moreover, the introduction of flood-proof sanitation facilities, such as raised latrines and septic tanks with impervious walls and lids, would be advisable to prevent contamination of and mitigate against the emergence of waterborne diseases in disaster-prone periods.

In summary, the current floods reveal the deeply seated public health vulnerabilities in this disaster-ridden region, and they are a grim reminder of the catastrophic impacts of climate change on our environment. Massively reducing the immediate risk of disease outbreaks requires immediate interventions. At the same time, longer-term strategies need to address the psychological and infrastructural wreckage. Through strengthening health systems, increasing community understanding and improving preparedness for disasters, Nepal can reduce the public health impact of future disasters and protect the well-being of its people.
